# Cytological and proteomic analyses of floral buds reveal an altered atlas of meiosis in autopolyploid *Brassica rapa*

**DOI:** 10.1186/s13578-019-0313-z

**Published:** 2019-06-17

**Authors:** Yan Yang, Fang Wei, Janeen Braynen, Xiaochun Wei, Baoming Tian, Gongyao Shi, Gangqiang Cao, Jiachen Yuan, Xiaowei Zhang

**Affiliations:** 1 School of Agricultural Sciences, Zhengzhou University, Zhengzhou, 450001 Henan People’s Republic of China; 2 School of Life Sciences, Zhengzhou University, Zhengzhou, 450001 Henan People’s Republic of China; 3 Institute of Horticultural Research, Henan Academy of Agricultural Sciences, Zhengzhou, 450002 Henan People’s Republic of China

**Keywords:** Meiosis, Proteome, Floral buds, Autopolyploid, *Brassica rapa*

## Abstract

**Background:**

Polyploidy is considered as a basic event in plant speciation and evolution in nature, and the cytological and proteomic profilings of floral buds at meiosis (FAM) would definitely contribute to a better understanding of the polyploid-associated effects during plant reproduction cycle.

**Results:**

Herein, the cytological investigations demonstrated that chromosome behaviors such as univalent and multivalent at prophase I, chaotic alignments at metaphase, aberrant segregation at telophase, were frequently observed during meiosis in autotetraploid *Brassica rapa*. The proteomic analysis showed a total of 562 differentially expressed proteins (DEPs) were identified in FAM between autotetraploid and diploid *B. rapa*. Notably, PARP2 and LIG1 related to base excision repair and BARD1 involved in recombination were significantly down-regulated in autotetraploid *B. rapa*, which indicated DNA repair pathway were more likely affected during meiosis in autotetraploid *B. rapa*. The functional analysis showed that DEPs assigned to “chromatin structure and dynamics”, “cell cycle control, cell division, chromosome partitioning” and “cytoskeleton” were preferentially up-regulated, which suggested a robust regulation of cell division in autotetraploid *B. rapa*. In combination with the floral RNA-seq data released, a number of DEPs were found positively correlated with their transcript abundance, but posttranslational modification of proteins might also play a role in regulating meiosis course after polyploidization.

**Conclusions:**

In general, this study provides a detailed cytology and proteome landscape of FAM between diploid and autotetraploid *B. rapa*, which definitely affords us a better understanding of uniformity and discrepancy of meiosis at the plant reproductive stage before and after polyploidization.

**Electronic supplementary material:**

The online version of this article (10.1186/s13578-019-0313-z) contains supplementary material, which is available to authorized users.

## Introduction

Polyploidy is a widespread and prominent process that plays an important role in the evolution of all angiosperm plants through genomic merging and doubling [1]. There are two main types of polyploids: autopolyploids and allopolyploids, and most polyploids have diploid ancestries [2]. Compared with the diploids, autopolyploids combine more than two sets of identical genomes in their nucleus [3]. They often survive in severe environments and trend to have larger cells, resulting in the enlargement of some organs, such as leaves and flowers [4–6]. Despite the widespread occurrence of polyploidy, it still remains controversial to explain the direct effects on plant reproduction process after genome doubling in the polyploid species [7–9].

Formation of a flower is considered as the most important stage for plant reproduction during the whole life cycle [10]. It is an indicator of transformation, from vegetative growth to reproductive growth and have a complex process that involves physiology, biochemistry and morphology. Meiosis occurs within the reproductive organs [11], which is a fundamental process in all sexual organisms that ensures fertility [12]. In addition to offering evolutionary and phenotypic diversity, polyploidy has also considerable impacts on the reproductive process in plants [13]. For instance, a higher percentage of abnormal chromosome behaviors during meiosis in pollen mother cells (PMCs) was frequently observed in polyploids [14, 15].

With the growing application of the omics approaches, transcriptomics has contributed a lot to the identification of genes involved in plant reproduction process. Especially, meiosis as a key pre-zygotic stage during plant reproductive life was investigated at the transcriptomic level in both diploid and polyploid plants [16–18]. In conjunction with transcriptomic analysis, proteomic analysis is increasingly used in plant functional genomic studies and has potential to provide a broader viewpoint at the level of proteins. Several proteome studies revealed the proteomic alternations underpinning key biological and physiological processes after polyploidization. For example, leaf proteome analyses in *Brassica oleracea* and *Arabidopsis thaliana* autopolyploids showed that expression of proteins was not significantly affected by the ploidy level in tetraploids [19, 20]. But the quantitative analyses of protein abundance in *Paulownia* and *Cassava* suggested that autotetraploid exhibited better photosynthetic characteristics and higher stress resistance [21, 22]. In the context of polyploidization, such a proteomic analysis is also becoming necessary to evaluate effects of polyploidy on plant reproduction in polyploid plants.

In the present work, the cytology and proteome of FAM were analyzed in autotetraploid *B. rapa* in comparison with its diploids, and a correlation between this proteomic profiling and mRNA transcripts was also investigated. Undoubtedly, these results would greatly increase our knowledge about the polyploidy-associated impacts on plant reproduction at cytological and proteomic levels in autopolyploid species in contrast with their diploid counterparts.

## Materials and methods

### Plant material and growth conditions

The diploid *B. rapa* seedlings was artificially induced with 0.2% colchicine treatment for 2 days. Chromosome counts and flow cytometry were used to identify the autotetraploids. All *B. rapa* plants used in this study were grown in the green house under 16 h light and 8 h dark photoperiod, at temperatures of 22 °C daytime and 18 °C night. The stage of FAM was determined by microscopic examination of the appearance and FAM with 1.0–1.5 mm (identified at meiosis) length were collected.

### Cytology

Inflorescences were collected and fixed in Carnoy’s solution (alcohol:glacial acetic acid, 3:1 v/v) overnight at RT and stored in 70% ethanol at 4 °C until use. The buds of proper size in 1.0–1.5 mm approximately were rinsed with distilled water (3 × 3 min) and citrate buffer (10 mM, pH 4.5) (2 × 5 min). Anthers removed from the floret using a dissecting needle under stero microscope and incubated in enzyme mix including pectolase (0.5% w/v) and cellulase (0.5% w/v) in citrate buffer for 4 h at 37 °C. The chromosome spreads were prepared as previously described [23] with minor modifications. The prepared slides were stained with 40 µg/mg PI solution for 5 min, and then observed with fluorescence microscope.

### Immunofluorescence

Inflorescences were collected and fixed in 4% (w/v) paraformaldehyde and the chromosome slides were prepared as previously described [23] with minor modifications. Each slide was blocked in 1% BSA in PBS for 60 min and then incubated overnight at 4 °C in a moist chamber with 50 μl anti-γH2AX polyclonal antibody (Trevigen 4418-APC-100) diluted 1:100 in blocking buffer (3% BSA in PBS). Slides were washed three times for 5 min in PBS solution and incubated for 2 h at 37 °C with goat anti-rabbit FITC secondary antibody. The chromosome slides were washed three times for 5 min in PBS and then air dried. Finally, slides were counterstained with 40 µg/mg PI solution in an antifade solution and observed with fluorescence microscope.

### Protein preparation

The FAM were firstly harvested and immediately frozen and kept in liquid nitrogen in three biological replicates until use. Sample was first grinded by liquid nitrogen, then the cell powder was sonicated three times on ice using a high intensity ultrasonic processor (Scientz) in lysis buffer (8 M urea, 2 mM EDTA, 10 mM DTT and 1% Protease inhibitor cocktail), followed by centrifugation at 20,000*g* at 4 °C for 10 min. The pellets were precipitated with cold 15% TCA for 2 h at − 20 °C, and then centrifugation at 4 °C for 10 min. The precipitate was redissolved in buffer (8 M urea, 100 mM TEAB, pH 8.0) and the protein concentration was determined with 2-D Quant kit according to the manufacturer’s instructions.

### Protein digestion and TMT labeling

For digestion, the protein solution was reduced with 10 mM DTT for 1 h at 37 °C and alkylated with 20 mM IAA for 45 min at room temperature in darkness. For trypsin digestion, the protein sample was diluted by adding 100 mM TEAB to urea concentration less than 2 M. Finally, the samples were digested for the first digestion overnight and for a second 4 h-digestion.

After trypsin digestion, peptide was desalted and vacuum-dried. The TMT labeling procedure was following the manufacturer’s protocol for 6-plex TMT kit. Briefly, one unit of TMT reagent (defined as the amount of reagent required to label 100 μg of protein) were thawed and reconstituted in 24 μl ACN. The peptide mixtures were then incubated for 2 h at room temperature and pooled, desalted and dried by vacuum centrifugation.

### HPLC fractionation

The sample was then fractionated into fractions by high pH reverse-phase HPLC using Agilent 300Extend C18 column (5 μm particles, 4.6 mm ID, 250 mm length). Briefly, peptides were first separated with a gradient of 2% to 60% acetonitrile in 10 mM ammonium bicarbonate pH 10 over 80 min into 80 fractions. Then, the peptides were combined into 18 fractions and dried by vacuum centrifuging.

### LC–MS/MS

The dry peptides were dissolved in 0.1% FA and directly loaded onto a reversed-phase pre-column (Acclaim PepMap 100, Thermo Scientific). Peptide separation was performed by a reversed-phase analytical column (Acclaim PepMap RSLC, Thermo Scientific). The dry samples were eluted in a column gradient mixture of solvent A/B. Using solvent B (0.1% FA in 98% ACN), gradients increased from 8 to 26% in over 22 min, then 26% to 40% in 12 min and finally climbed to 80% in 3 min. The column was maintained at a constant flow rate of 400 nl/min on an EASY-nLC 1000 UPLC system.

The peptides were subjected to NSI source followed by tandem mass spectrometry (MS/MS) with the use of Q Exactive TM plus (Thermo) coupled online to the UPLC. Intact peptides were detected in the Orbitrap at a resolution of 70,000. Such peptides were selected for MS/MS using NCE setting as 30. In addition, ion fragments were detected in the Orbitrap at a resolution of 17,500. A data-dependent procedure that alternated between one MS scan followed by 20 MS/MS scans was applied for the top 20 precursor ions above a threshold ion count of 1E4 in the MS survey scan with 30.0 s dynamic exclusion. The electrospray voltage applied was 2.0 kV. Automatic gain control was used to prevent overfilling of the orbitrap; 5E4 ions were accumulated for generation of MS/MS spectra. For MS scans, the m/z scan range was 350 to 1800. Fixed first mass was set as 100 m/z.

### Protein identification

The resulting MS/MS data was processed using MaxQuant software. Trypsin/P was specified as cleavage enzyme allowing up to two missing cleavages. Mass error was set to 10 ppm for precursor ions and 0.02 Da for fragment ions. Carbamidomethylation on Cys was specified as fixed modification and oxidation on Met was specified as variable modifications. False discovery rate (FDR) thresholds for protein, peptide and modification site were specified at 1%. Minimum peptide length was set at 7. For quantification method, TMT-6-plex was selected. The fold changes were used to identify up-regulated and down-regulated proteins with a > 1.5 or < 2/3 (p < 0.05).

### Bioinformatic analysis

Functional analysis of the identified proteins was conducted using Gene Ontology (GO) annotations from the UniProt-GOA database (http://www.ebi.ac.uk/GOA/). Converting identified protein ID to UniProt ID and mapping to GO IDs by protein ID. If the identified proteins were not annotated by UniProt-GOA database, the InterProScan soft would be used to annotated protein’s GO functional based on protein sequence alignment method. The proteins were categorized according to their biological processes, molecular functions and cellular localizations. The proteins were further analyzed using the Eukaryotic Orthologous Groups (KOG) database (http://www.ncbi.nlm.nih.gov/COG/).

### RNA preparation and quantitative RT-PCR

TriZol reagent was used to extract total RNA of FAM and then precipitated with isopropanol. 1 µg of the purified sample was constructed the first-strand cDNA using SuperScript III Reverse Transcriptase (Invitrogen) kit. Primers were designed by Primer premier 5.0 (PREMIERThe RNA samples from Biosoft) and checked with Blast primer design tool (NCBI; Additional file 1: Table S1). The qRT-PCR reactions were performed with 1× SYBR master kit and repeated in three independent experiments.

### Correlation analysis between mRNA seq data and identified proteins

The RNA-seq data were collected from the NCBI Sequences Read Archives (SRA) according to the Accession Number SRP104015. The genome data of *B. rapa* was downloaded from *Brassica* database (http://brassicadb.org/brad/). The correlation analysis for the identified proteins and mRNA transcripts were performed according to the genome annotations using the circlize R package.

## Results

### Determination of *B. rapa* autotetraploids

**Fig. 1:**
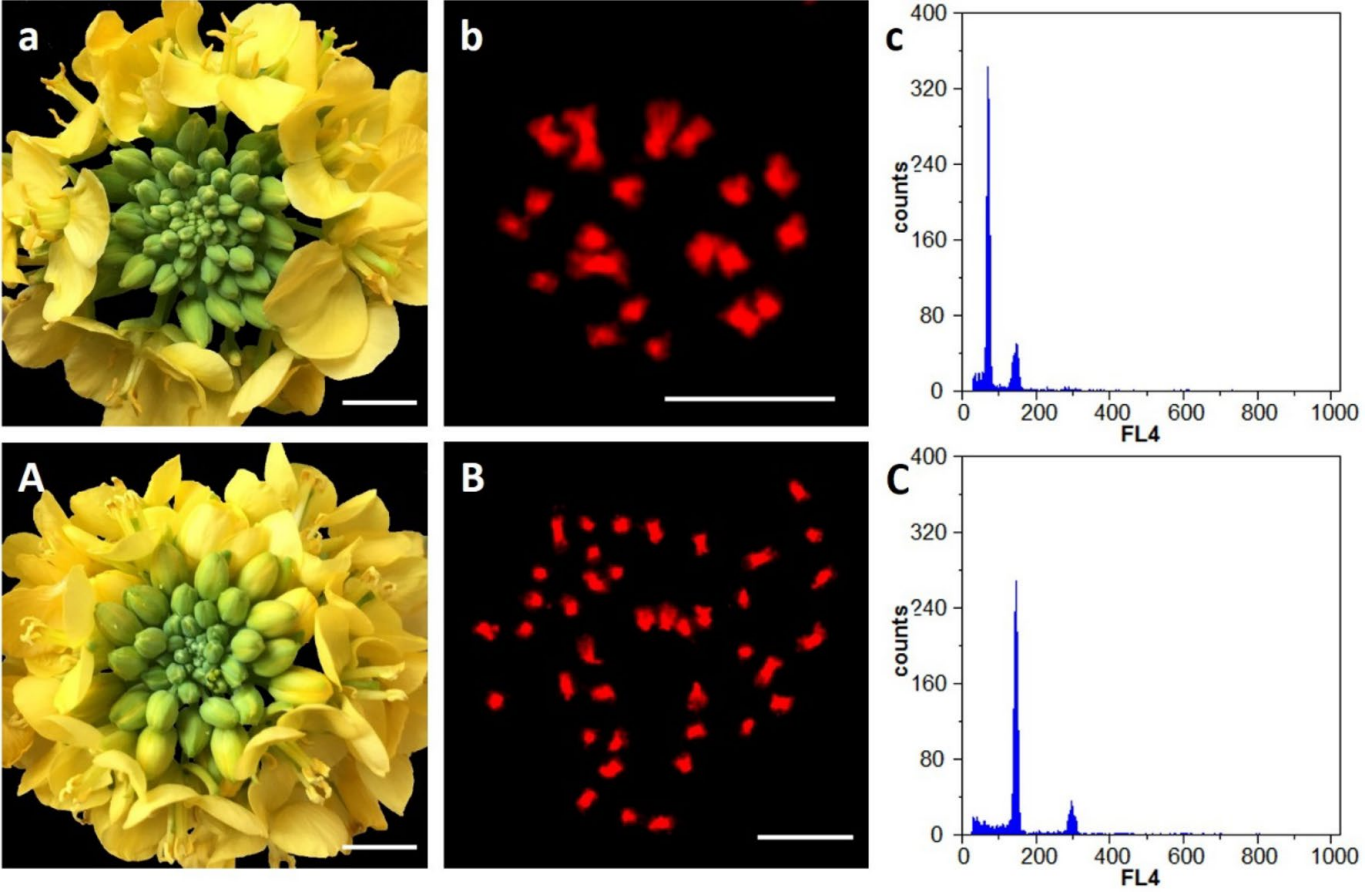
Flower phenotype, chromosome number and flow cytometric analysis of the colchicine-induced *B. rapa* plants. **a**, **A** Flower of diploid *B. rapa* and the enlarged flower of the treated *B. rapa* plant. Bar = 1 cm; **b**, **B** exactly 20 chromosomes in diploid *B. rapa* and 40 chromosomes counted in autotetraploid *B. rapa*. Bar = 10 μm; **c**, **C** the altered peak distribution of nuclear DNA content in autotetraploid in comparison with diploid *B. rapa*

As shown in Fig. 1, a cluster of gigantism flower buds were obviously obtained in the colchicine-induced *B. rapa* plants compared with diploids, and the flow cytometric analysis showed that an increase of DNA contents were also detected in these induced plants. Moreover, the chromosome counting indicated the induced *B. rapa* plants exactly gained 40 chromosomes after chemical treatment in comparison with diploids.

### Chromosome behaviors during meiosis in autotetraploid *B. rapa*

**Fig. 2:**
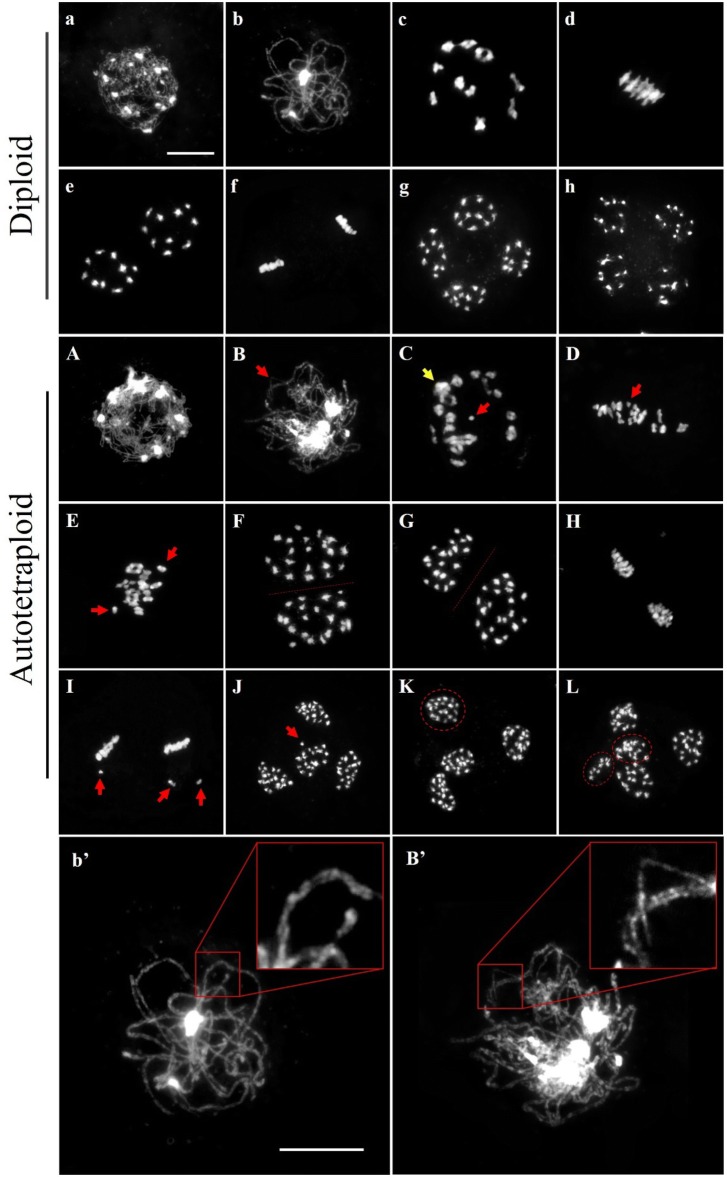
Aberrant chromosome behaviors during meiosis in autotetraploid *B. rapa*. Normal meiosis from leptotene to telophase II in diploid *B. rapa*. **a** Leptotene, the chromosomes were shown as thin threads. **b** Pachytene, homologous chromosomes were completed synapsis. **c** Diakinesis, chromosomes moderately condensed as bivalents, **d** metaphase I, bivalents aligned on the equatorial plate. **e** Anaphase I, homologous chromosomes separated and move toward the opposite poles. **f** Metaphase II, two groups of condensed chromosomes at equatorial plate. **g** Anaphase II, chromatids separated each other and move toward the opposite poles of tetrad. **h** Telophase II, tetrads formation. Abnormal meiosis in autotetraploid *B. rapa*. **A** Leptotene, chromosome morphology was similar to diploid *B. rapa*. **B** Abnormal pachytene, some chromosomes are not closely juxtaposed with another (red arrow) suggests that synaptonemal complex is incomplete. **C** Abnormal diakinesis, multivalent (yellow arrow) and univalent (red arrow) were frequently observed. **D** Abnormal metaphase I, variable numbers of univalent (red arrow) aligned chaotically on the equatorial plate. **E** Abnormal metaphase I, asynchrony separation of chromosomes (red arrows) were observed. **F** Anaphase I, homologous chromosomes separated (20/20) and move toward the opposite poles **G** abnormal anaphase I, homologous chromosomes separated asymmetrically (19/21). **H** Metaphase II, chromosomes aligned at the equator in each cell. **I** Abnormal metaphase II, misaligned chromosomes (red arrows) were oriented away from the equatorial plate. **J** Abnormal anaphase II, lagged chromosome (red arrow) segregated to the poles. **K** Abnormal anaphase II, unbalanced tetrads with 19 chromosomes (dotted oval). **L** Abnormal anaphase II, different kinds of meiosis products with pentad. Magnification of homologous chromosomes pairing and synapsis at pachytene. **b**′ Full synapsis and pairing in diploid *B. rapa*. **B**′ partial synapsis and pairing in autotetraploid *B. rapa*. Red box indicates the zoom-in of configuration. Bar = 10 μm

The chromosome behaviors of PMCs including synapsis, pairing and segregation during meiosis was evaluated in diploid and autotetraploid *B. rapa* (Fig. 2). Homologous chromosomes were completely synapsed at pachytene, paired as bivalents at diakinesis, orderly aligned at equatorial plate in metaphase, and equally segregated at anaphase I and anaphase II during meiosis in diploid *B. rapa* (Fig. 2a–h). Meiosis in autotetraploid *B. rapa* was also similar to original diploid *B. rapa*, but the pairing and segregation of chromosome in autotetraploid *B. rapa* was different from the diploid *B. rapa*. In comparison with full synapsis and pairing in diploid *B. rapa* (Fig. 2b′), homologous chromosomes are partially synapsed and paired at pachytene by the presence of more than two sets of homologous chromosomes in autotetraploid *B. rapa* (Fig. 2B′). The chromosomes were partially tangled at later diakinesis (Fig. 2C), formed as univalent at metaphase (Fig. 2D, E, H, I), and segregated unequally with a few lagged chromosomes at anaphase in autotetraploid *B. rapa* (Fig. 2F, G, J–L).

Moreover, a statistic analysis was conducted to observe the abnormal chromosome behavior between the diploid and autotetraploid *B. rapa*. According to the results, the average frequencies of chromosomal abnormalities in autotetraploid *B. rapa* were higher than diploid *B. rapa* during meiosis process (Additional file 2: Fig. S1). Chromosome multivalents and univalents were occurred in 50.6% observed PMCs at metaphase I (79 cells observed). Approximately, 30.1% of PMCs produced the abnormal separation with unbalanced dyad (113 cells), and 25.8% observed PMCs consist of chromosome straggling at metaphase II (62 cells). Chromosome lagging and different kinds of meiosis products were detected at anaphase II in 37.8% PMCs (156 cells) in autotetraploid *B. rapa*. Therefore, these results indicated that the meiosis course was affected in autotetraploid *B. rapa* after polyploidization.

We also sought the dynamic process of meiotic DSB formation and repair by γ-H2AX during prophase I (Additional file 2: Fig. S2). In diploid *B. rapa*, numerous diffuse γH2AX signals were detected throughout the chromatin at zygotene (Additional file 2: Fig. S2a) and increased at pachytene (Additional file 2: Fig. S2b). Following diplotene and diakinesis, the γH2AX foci disappeared gradually (Additional file 2: Fig S2c–d), suggesting that the DSBs were repaired normally. However, strong γH2AX signals were observed until diakinesis in autotetraploid (Additional file 2: Fig. S2A–D). Thus, the observations indicate that DSBs formation occurred normally but DSBs repair progression failed at subsequent prophase I in autotetraploid.

### Characterization of FAM proteomics data

A total of 271,286 spectrums were generated from the quantitative proteomics analysis of FAM from both diploid and autotetraploid *B. rapa*. The analysis identified 74,786 spectrums that matched to known spectrums. Among them, 46,120 peptides were found and 35,315 unique peptides could be matched to 9298 proteins. As a result, 7636 proteins were quantified (Additional file 2: Fig. S3a), which consisted of the range from 1 to 65 peptides (Additional file 2: Fig. S3b). The majority of these proteins were larger than 10 kDa and their molecular weights covered a wide range (Additional file 2: Fig. S3c). Most of the proteins had good peptide coverage; 57% had more than 10% coverage, and 29% had 20% coverage (Additional file 2: Fig. S3d). The reproducibility of the proteomic analysis is shown in Additional file 2: Fig. S4. These results indicate that the proteomics analyses were reliable and reproducible.

In this analysis, DEPs were defined as proteins that were quantified with a greater amount no less than 1.5-fold in relative abundance and a p < 0.05, and a total of 562 DEPs were thus identified between autotetraploid *B. rapa* and diploid *B. rapa*. Among which 267 DEPs were up-regulated and 295 DEPs were down-regulated in the flower buds of autotetraploid *B. rapa* in comparison with its diploid (Additional file 1: Table S2). Among these DEPs identified, six proteins were newly identified and two of them were not yet described previously, which remain functionally unknown (Additional file 1: Table S2).

### GO analysis of identified proteins and meiosis-related proteins

**Fig. 3:**
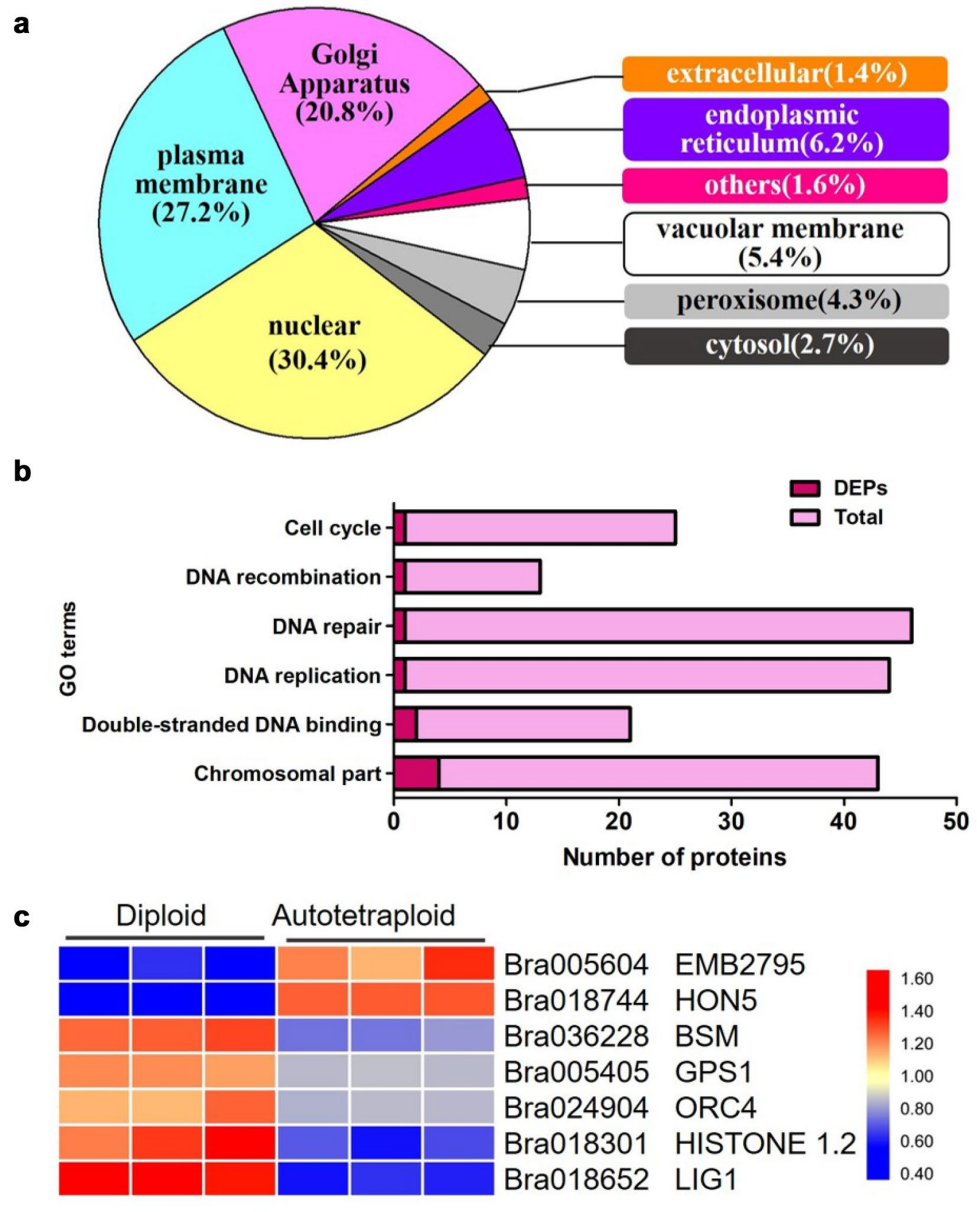
Subcellular localization and GO classification of meiosis in autotetraploid *B. rapa*. **a** Subcellular localization of DEPs in autotetraploid *B. rapa*. **b** Number of identified proteins and EDPs in meiosis-related classification. **c** Expression patterns of 7 DEPs in meiosis classification in diploid and autotetraploid *B. rapa*

GO classification was firstly performed for all the identified proteins in FAM of both diploid and autotetraploid *B. rapa*. As a result, approximately 7037 known proteins could be generally assigned to three different terms: (1) in the cellular component category, cell (1361) was enriched with the largest number of proteins; (2) in respect to molecular function, binding (4215) and catalytic activity (3493) were over-represented; (3) in regard to biological process, metabolic process (3301), cellular process (2750) and single-organism process (1889) were among the three largest groups (Additional file 2: Fig. S5a). These results indicated that the identified proteins are involved in a wide range of biological processes in the flower bud development of *B. rapa*. The subcellular location of the quantitated proteins was also analyzed (Additional file 2: Fig. S5b). The proteins were mostly distributed in chloroplast (3068), and followed group was located in nucleus (2402), and cytosol (2099). The results show that the proteins distributed in a variety of organelles. However, the subcellular distributions of DEPs were much different from all the identified proteins (Fig. 3a), with the largest proportion assigned to nucleus (171), and followed by plasma membrane (153), and Golgi apparatus (117), which indicated the proteins in these organelles might be susceptible after polyploidization. Considering critical players associated with chromosomes behaviors during meiosis were probably located in nucleus, the identification of proteins in this process could lead us to the molecular pathway controlling meiosis at the protein level.

Notably, 154 proteins were assigned to meiosis related categories, including chromosomal part, double-stranded DNA binding, DNA replication, DNA repair, DNA recombination and cell cycle (Fig. 3b). However, only seven proteins were differentially expressed in autotetraploid *B. rapa* compared with diploid *B. rapa* (Fig. 3c), which implied the expression of meiosis-related proteins were mostly independent of ploidy level. Among seven DEPs, five DEPs were down-regulated and two DEPs were up-regulated in the FAM of autotetraploid *B. rapa* in comparison with its diploids. Two proteins EMB2795 (Bra005604) and HON5 (Bra018744) related to cell division and chromosome condensation were up-regulated in autotetraploid *B. rapa*. These results implied that cell division process was highly active in autotetraploids. However, HISTONE1.2 (Bra018301), ORC4 (Bra024904) and LIG1 (Bra018652) associated with DNA recombination, DNA replication and DSBs repair showed a down-regulation, which indicated that DSBs repair may be accomplished slightly in autotetraploid *B. rapa*. In addition, 12 DEPs (TPI, CPK1, PEN2, PGK1, SRF2, MKK4, Bra031951, Bra016114, Bra031535, Bra035078, Bra030815, Bra022240) associated with phosphorylation were also found out (Additional file 2: Fig. S6), which might indicate a post-translational phosphorylation event to regulate meiosis-related proteins in FAM of autotetraploid *B. rapa.*

### KOG analysis of identified proteins and meiosis-related proteins

**Fig. 4:**
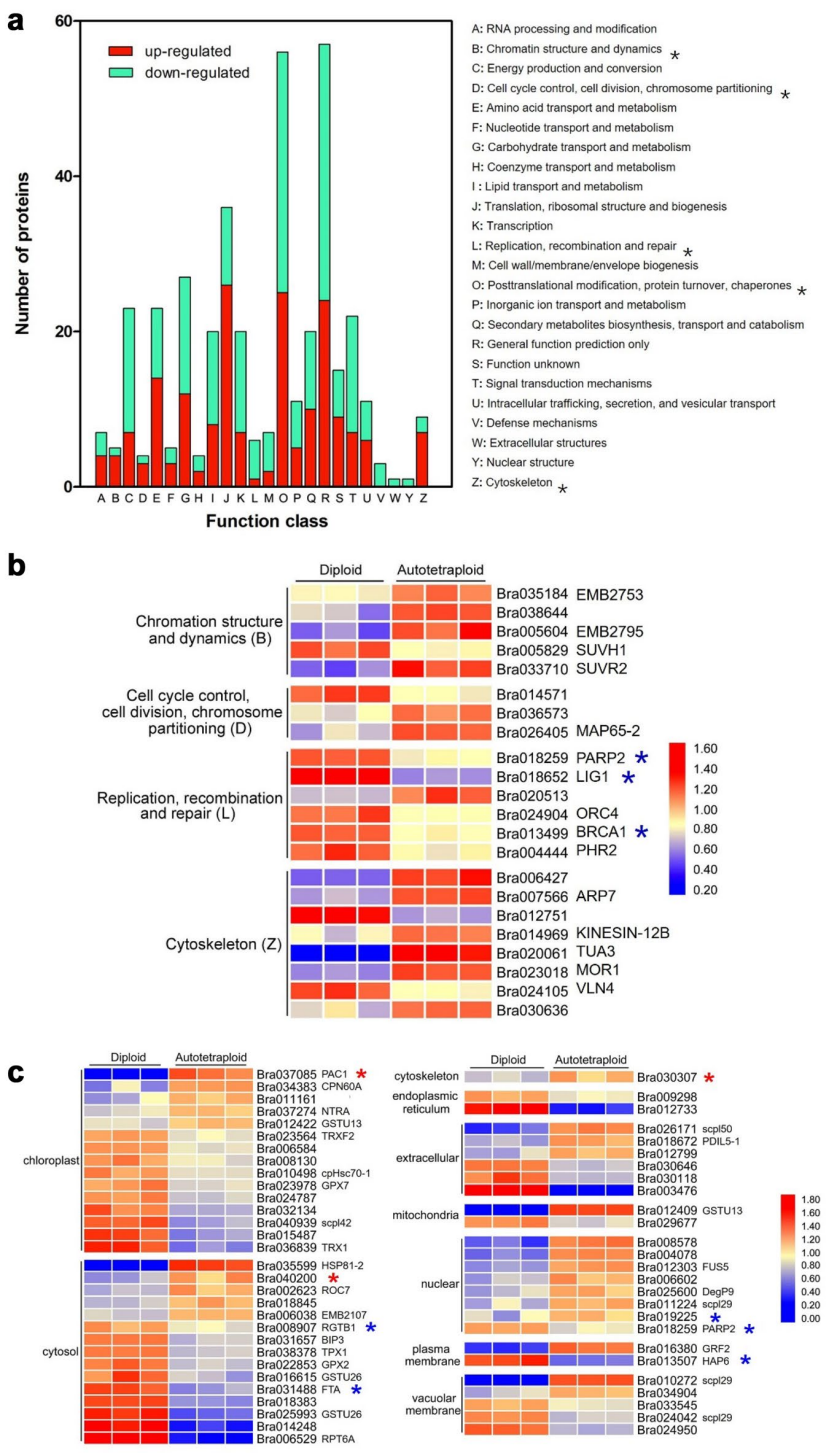
KOG classification and expression patterns of DEPs in autotetraploid *B. rapa*. **a** The asterisks represent functional classification of putative meiosis-related proteins. **b** Expression patterns of 22 DEPs in meiosis category in diploid and autotetraploid *B. rapa*. **c** Expression patterns of 56 DEPs in posttranslational modification in diploid and autotetraploid *B. rapa*

To understand the function of identified proteins, 6837 proteins were classified into 25 categories using the KOG database. The KOG clusters revealed that the overrepresented functional categories were “General function prediction only (R)” and “Posttranslational modification, protein turnover, chaperones (O)”, which represented 14% and 12% of the identified proteins, respectively (Additional file 2: Fig. S7). In addition, clustering of KOG was also performed for the DEPs, and 353 DEPs were divided into 24 categories (Fig. 4a). The DEPs identified with functions in “posttranslational modification, protein turnover, chaperones (O)” and “translation, ribosomal structure and biogenesis (J)” were over-represented, which suggested that the function regulation could occur at the transcription (mRNA), translation (protein) and post-translation (protein modification) levels after polyploidization. Additionally, several proteins involved in “defense mechanisms (V)”, “extracellular structures (W)” and “nuclear structure (Y)” were solely down-regulated in autotetraploid *B. rapa*, which were definitely needed for future analyses for their possible roles in plant reproductive process after polyploidization.

To characterize protein expression profiling associated with meiosis, a total of 22 DEPs were involved in “cell cycle control, cell division, chromosome partitioning (D)”, “cytoskeleton (Z)”, “chromosome structure and dynamics(B)” and “replication, recombination and repair (L)” (Fig. 4b). Among these, most proteins associated with “cell cycle control, cell division, chromosome partitioning (D)”, “cytoskeleton (Z)” and “chromosome structure and dynamics (B)” were up-regulated. EMB2795 (Bra005604), MAP65-2 (Bra026405), KINESIN-12B (Bra014969), ARP7 (Bra007566), TUA3 (Bra020061) and MOR1 (Bra023018) showed up-regulation in autotetraploid *B. rapa* (Fig. 4b), which likely indicated a more robust activity of cell division in autotetraploid *B. rapa*. However, proteins associated with specific repair and recombination pathway were down-regulated. Two proteins PARP2 (Bra018259) and LIG1 (Bra018652) related to base excision repair and nuclear excision repair, and protein BARD1 (Bra013499) involved in homologous recombination displayed down-regulation in autotetraploid *B. rapa* (Fig. 4b), which implied that the DNA repair pathway was probably affected during meiosis in autotetraploid *B. rapa*. In addition, the interaction of 22 meiosis-related proteins were predicted using the STRING, and the results showed that 10 proteins constituted a regulatory network (Additional file 2: Fig. S8), and particularly the up-regulated protein MOR1 interacted with four up-regulated proteins, which may make for the functional enhancement.

To better understand the modification of the DEPs, we further assessed the protein expression profiling associated with posttranslational modification according to KOG category. 56 proteins, including protein folding, proteolysis, and metabolic process, were found. Most proteins were distributed in chloroplast, cytosol and nucleus, but only proteins in nucleus displayed significant up-regulation (Fig. 4c). Among these, three ubiquitin associated protein catabolic process (PAC1, Bra030307, Bra040200 and Bra040200) showed up-regulation in autotetraploid *B. rapa* (Fig. 4c), which indicated that the ubiquitin-dependent protein catabolic process plays a role in regulating meiosis course and result in a faster turnover of protein in autotetraploid *B. rapa* after polyploidization. Modifications including protein glycosylation, geranylgeranylation and prenylation were also detected and mostly down-regulated (PARP2, HAP6, RGTB1, FTA, Bra019225). Interestingly, two down-regulated proteins RGTB1 (Bra008907) and PARP2 (Bra018259) were also related to pollen development and DSBs repair. These results indicated that the meiosis was also probably regulated by posttranslational modification in autotetraploid *B. rapa* after polyploidization.

### Correlation between identified DEPs and mRNA transcripts

**Fig. 5:**
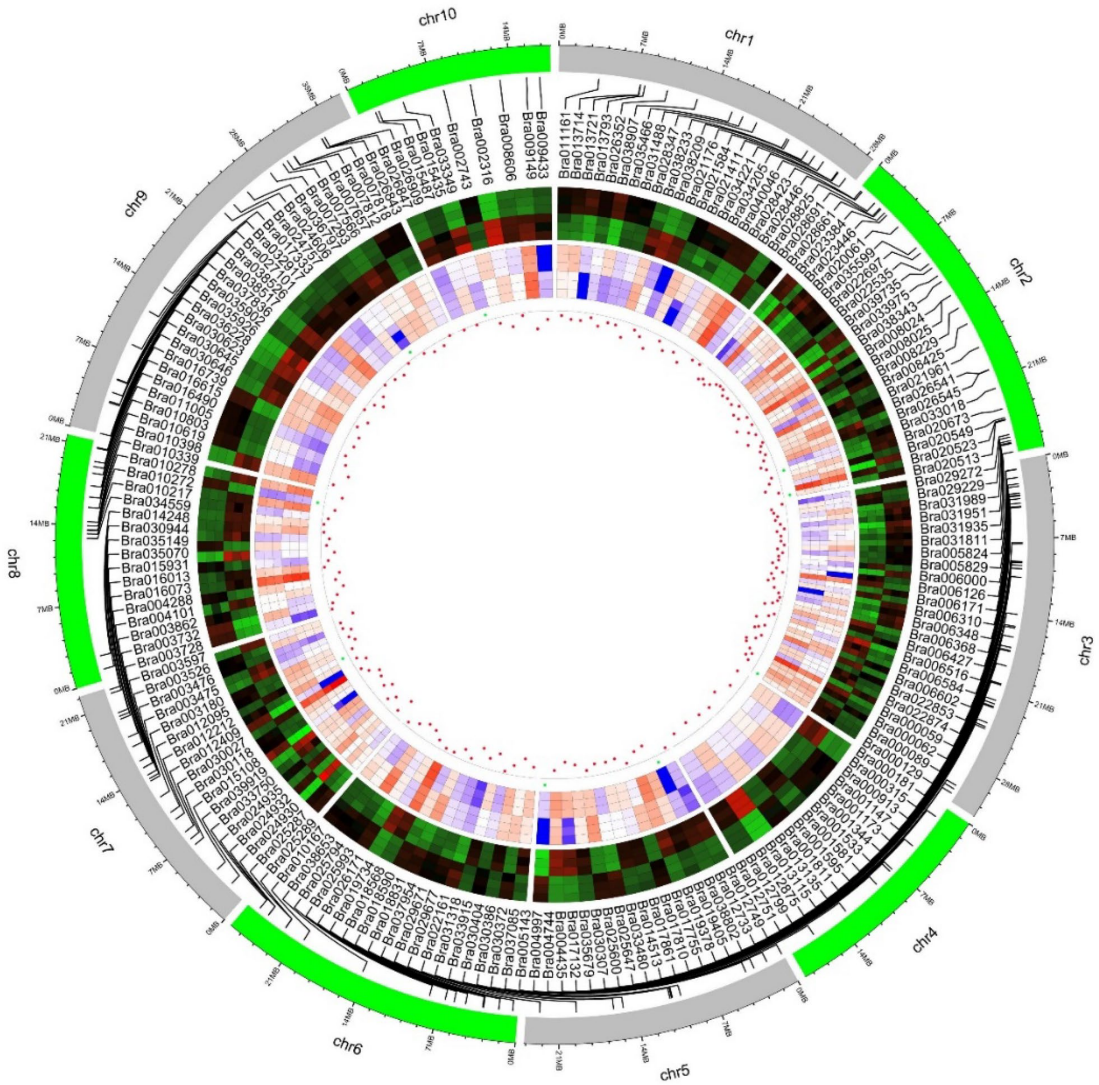
Correlation analysis for the identified 193 DEPs with their transcriptome. In the circular plot, the correlation of expression patterns were surrounded by 10 chromosomes. Protein characterization between diploid and autotetraploid were shown in the rings of blocks (red and green) adjacent to the chromosome. The gene expression pattern was shown in the following rings (red and blue), the dots in the inner are the results of correlation (red dots indicate a positive correlation and vice versa). 11 proteins in the scaffold regions were not shown

To compare changes in protein abundance with alterations in the mRNA transcripts, we matched all identified proteins with mRNA transcripts derived from a previous transcriptome analysis in autotetraploid *B. rapa*. In general, these identified 562 DEPs were well matched with their encoding genes at the mRNA level. Among these, 194 differentially expressed genes (DEGs) showed noteworthy changes corresponding with their protein abundance, with 104 DEGs up-regulated and 90 DEGs down-regulated respectively. Conversely, we also found a small fraction of DEGs in the mRNA levels, displayed an opposition in their protein abundance (Fig. 5, Additional file 1: Table S3). In addition, 358 DEPs with changes in abundance showed no obvious changes in their mRNA expression levels (Additional file 1: Table S4, Additional file 2: Fig. S9). Interestingly, among 22 meiosis-related proteins, only six proteins were consisted with mRNA transcripts level. These results indicated that in addition to the regulated at the equivalent level from mRNA transcripts to proteins, the posttranslational modifications might also play roles in regulating function of meiosis related proteins in autotetraploid *B. rapa* after polyploidization.

**Fig. 6:**
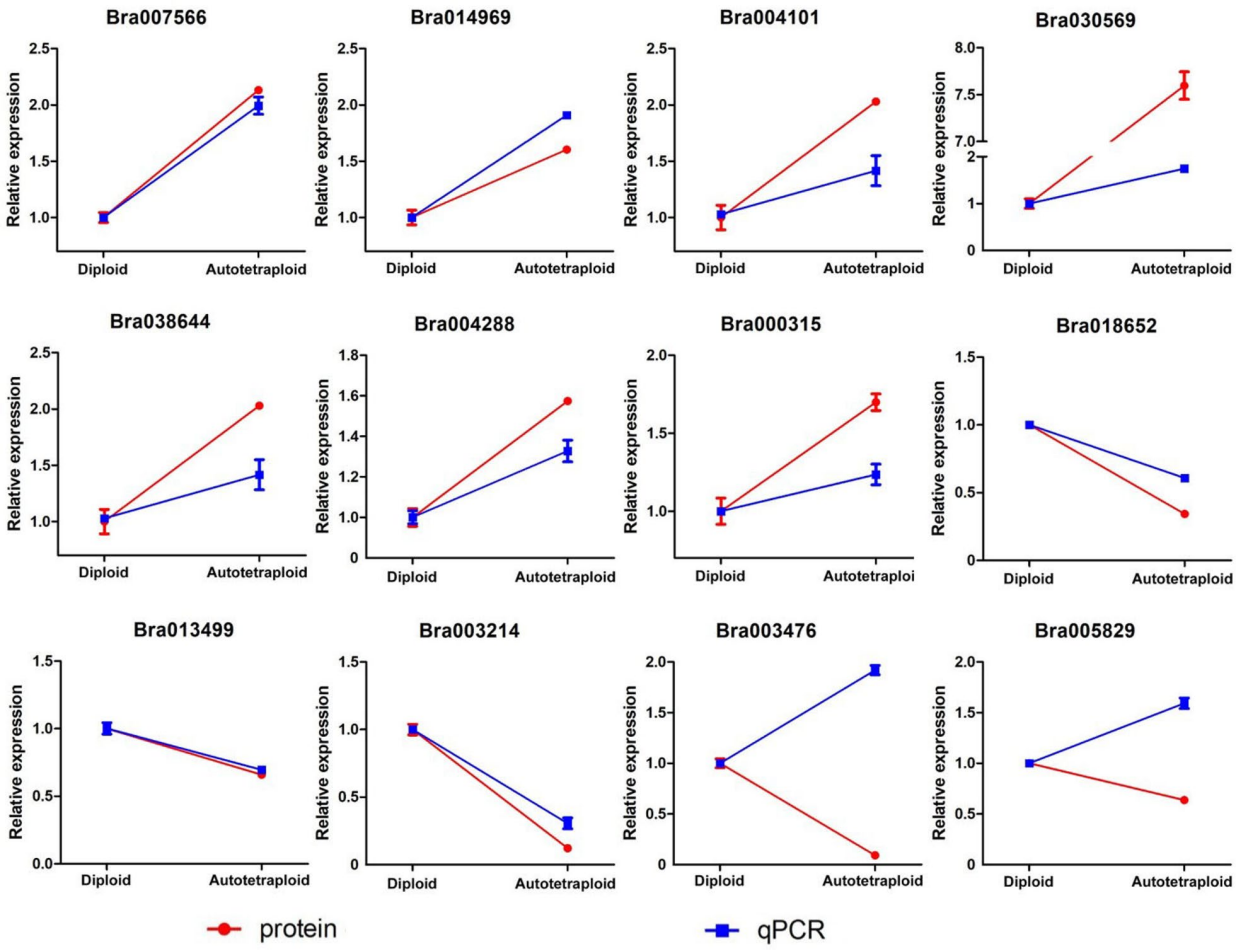
Quantitative RT-PCR analysis of differentially expressed proteins in autotetraploid *B. rapa*. The beta-actin was used as an internal reference for normalization

To confirm abundance of differentially expressed proteins at the transcriptional level, the expression of genes encoding their corresponding proteins was analyzed with qRT-PCR. The results showed that the transcript number for most selected genes coordinated well with their protein abundance in both diploid and autotetraploid *B. rapa*, including down-regulated meiosis-related proteins LIG1 (Bra018652) and BRCA1 (Bra013499), though a slight discrepancy was also observed between protein abundance and mRNA expression (Fig. 6). These results indicated the function of biological molecules were coordinated well at both transcriptional and protein levels, and also likely occurs at posttranscriptional and/or posttranslational processes.

## Discussion

### Down-regulation of meiosis-related proteins probably involved in altered meiosis in autopolyploid *B. rapa*

**Fig. 7:**
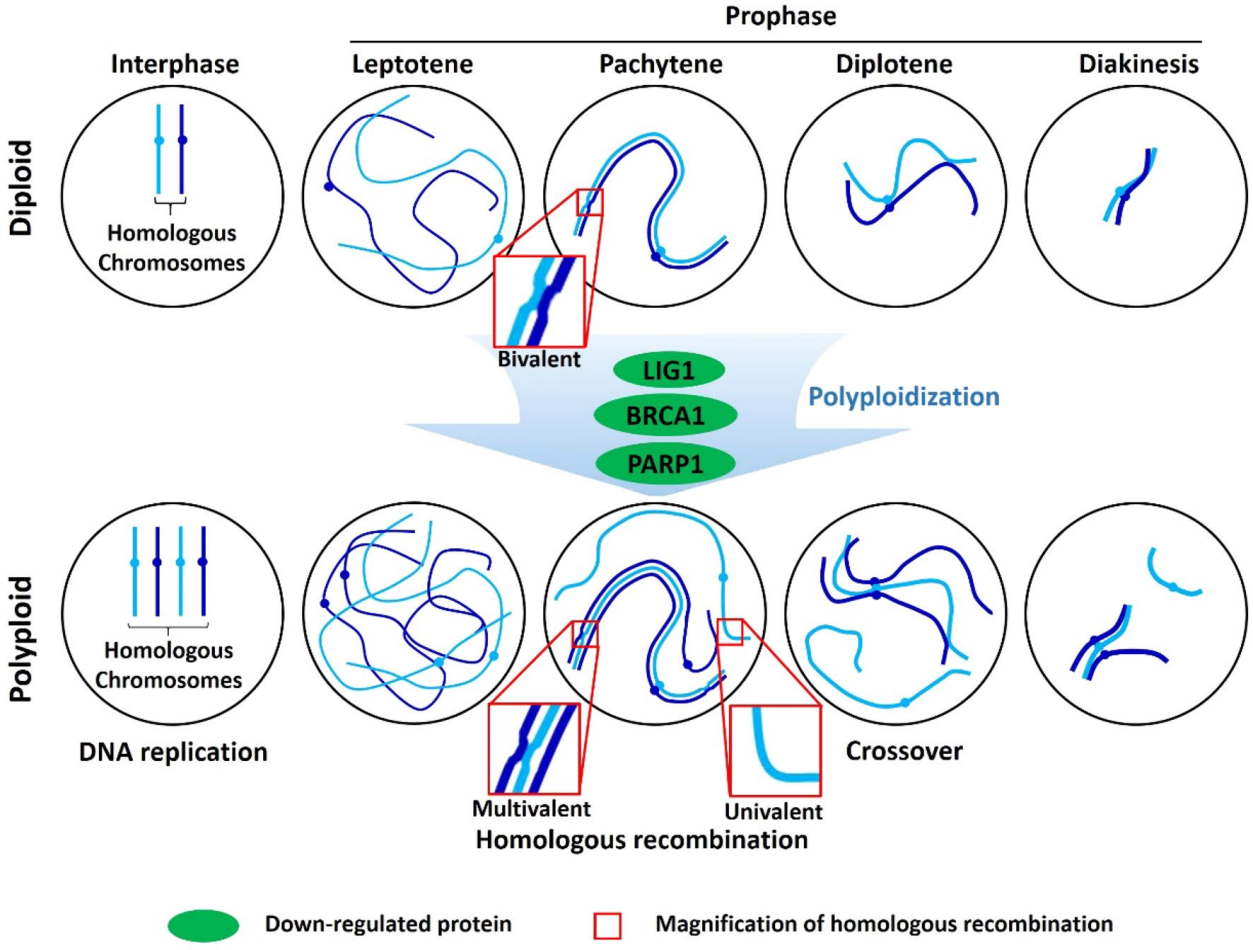
Early meiotic events with three meiosis-related EDPs in autopolyploid. Meiosis is preceded by DNA replication. Meiotic chromosomes start to condense and search for homologue during leptotene. Chromosome synapsis as synaptonemal complex (SC) during pachytene. The SC forms between pairs of homologous chromosomes (red box) in diploids. Crossovers are formed when SC disassemble at diplotene. Bivalents are visualized at diakinesis when chromosomes recondense. But SC can form between more than two homologues (red box) because of three down-regulated proteins, result in univalent and multivalent in autotetraploids

The polyploidy-associated effects have been extensively reviewed in expression patterns, environmental stress and chromosomal behavior, to explain changes between the established polyploids and diploids [24, 25]. It has been reported that polyploids need to overcome genetic instability occurring during meiosis and environmental pressures to survive [26, 27]. In the previous studies, meiosis-related genes associated with meiotic repair of DNA double-strand breaks (DSBs) were significantly down-regulated in autotetraploids compared with diploid *B. rapa* [18]. *DMC1* and *PAIR2*, related to homologous pairing and synapsis [28, 29], *MTOPVIB* and *MOF*, essential for meiotic DSB formation and telomere bouquet formation [30, 31], were found to be down-regulated in autotetraploid rice [14, 15]. Based on our protein expressions, DMC1 and ASY1 (*ASY1* in *B. rapa* is an ortholog with *PAIR2* in rice) showed stable expression in diploid and autotetraploid *B. rapa*. In addition, *lig1* RNAi-silenced lines showed very reduced Base excision repair in *Arabidopsis* [32]. And we also found that PARP1 (Bra018259), LIG1 (Bra018652) and BARD1 (Bra013499) involved in repairing damaged DNA and homologous recombination were down-regulated in autotetraploid *B. rapa*. These down-regulation of proteins indicated that the recombination pathway for meiotic DSB repair was probably handicapped in autotetraploid *B. rapa*, which may increase the percentage of abnormal synapsis and number of univalent at diakinesis and metaphase in autotetraploid *B. rapa* (Fig. 7).

Higher ploidy level was associated with increased flower size [13, 33] and the size of plant organs depends upon the regulation of cell division and cell expansion [34–36]. Flower size and reproductive organs have been increased vigorously due to the increasing of cell size in autotetraploid plants [13, 37]. According to the KOG category, some proteins involved in cell division and replication tended to be differentially expressed in autotetraploids. For instance, EMB2795 (Bra005604), and MAP65-2 (Bra026405), KINESIN-12B (Bra014969), TUA3 (Bra020061), which play important roles in the process of cell division in *B. rapa*, turned out up-regulated in autotetraploid compared with its diploids. These findings suggest that the polyploidization may result in increase of cell division activities and provide the driving power for phenotype variation during development process.

### FAM proteome was partially correlated with transcriptome in autotetraploid *B. rapa*

To interpret biological processes, it is essential to investigate the correlation of protein abundance and mRNA transcript levels. In general, a low correlation between protein abundance and mRNA expression levels is more often observed. Numerous reports have suggested that transcript accumulation is not always conveyed to the final product protein. In the resynthesized *B. napus*, most of the DEPs were not explained by transcriptional changes, and the regulation of DEPs was more likely governed by post-transcriptional modifications [38]. In Japanese apricot flower buds, the expression pattern of proteins was inconsistent with the corresponding genes after GA_4_ treatment, which indicated a negative correlation between mRNA and protein abundance [39]. In *A. thaliana*, down-regulated proteins were not closely correlated with changes in the corresponding mRNAs under phosphate-deficient and low temperature stress conditions [40, 41]. The percentage of DEPs that matched previously reported DEGs was also relatively low between autotetraploid and diploid in *A. thaliana* and *paulownia* [20, 42]. Meanwhile, we also found a small fraction of DEGs in the mRNA levels, displayed an opposition in their protein abundance. And the expression difference of genes were not translated into expression difference of proteins may because of posttranscriptional regulation and/or posttranslational modification [43].

Extensive protein phosphorylation and ubiquitination was revealed during anther development in many organisms [44, 45]. Ubiquitination is a post-translational modification process that plays a central role in protein degradation in cell division, including meiotic cell cycle progression and recombination, suggesting an important post-translational modification affecting protein activity [45, 46]. Protein phosphorylation is a dynamic and reversible post-translational modification that has important biological roles in reproductive development, and it has been shown to affect meiosis in yeast and rice [44, 47]. In this study, our proteomic also identified some proteins involved in phosphorylation (TPI, CPK1, PEN2, PGK1, SRF2, MKK4, Bra031951, Bra016114, Bra031535, Bra035078, Bra030815, Bra022240) and ubiquitin associated catabolic process proteins (UFD1, Bra040200 and Bra037085),which can facilitate studies to reveal functional and regulatory mechanisms at protein modification level in plant reproduction in polyploid.

Notably, a strong concordance between changes in mRNA and protein translation was also observed among different studies and different plant tissues. In fission yeast, the mRNAs induced under oxidative stress strongly correlated with increased protein expression [48]. Most of the genes and corresponding proteins that exhibited the same pattern at the transcriptional and translational levels in autotetraploid *paulownia* [42]. At the mid stage of male-sterile flower, when hypertrophy of the tapetum first becomes evident, the transcript and protein levels of SOD are high in garlic [49]. Similarly, we found that 194 DEGs and corresponding DEPs exhibited the same tendency at transcriptional and translational levels, which might imply that changes in transcript levels may be one of the determinants for changes in protein abundance in regulating the floral buds of autotetraploid *B. rapa*.

## Conclusion

In the present work, this study integrates cytological and proteomic profilings alteration of FAM in diploid and autotetraploid *B. rapa* to understand the polyploid-associated effects during plant reproduction cycle. The cytological study showed that aberrant chromosome behaviors were frequently observed in FAM of autotetraploid *B. rapa*. The proteomic analysis indicated a robust regulation of cell division in autotetraploid *B. rapa*. Simultaneously, DNA repair pathway were more likely affected during meiosis in autotetraploid *B. rapa*. In addition, posttranslational modification of proteins might also play a role in regulating meiosis course after polyploidization. In general, this study is a valuable resource for understanding the uniformity and discrepancy of meiosis at the plant reproductive stage before and after polyploidization.

## Additional files

**Additional file 1: Table S1.** Primer sequences for quantitative RT-PCR analysis of candidate proteins. **Table S2.** DEPs identified in autotetraploid *B. rapa*. **Table S3.** The identified 204 DEPs with correlated DEGs in autotetraploid *B. rapa*. **Table S4.** The identified 358 DEPs with no change in the transcript level in autotetraploid *B. rapa.*

**Additional file 2: Fig. S1.** Statistics of abnormal meiotic course in autotetraploid *B. rapa*. **Fig. S2.** Immunolocalization of γH2AX in diploid and autotetraploid *B. rapa*. **Fig. S3.** Characterization of proteomics data. **Fig. S4.** Reproducibility analysis of three biological replicates by Pearson correlation coefficient. **Fig. S5.** Gene ontology classification and subcellular localization of identified proteins. **Fig. S6.** Expression patterns of 12 DEPs associated with phosphorylation in diploid and autotetraploid *B. rapa*. **Fig. S7.** KOG function classification of identified proteins. **Fig. S8.** Predicted protein–protein interaction network of meiosis-related DEPs in autotetraploid *B. rapa*. **Fig. S9.** Correlation analysis for the identified 305 DEPs and their transcriptome.

## Data Availability

All the data is contained in the manuscript.

## Abbreviations

DEPs: differentially expressed proteins
FAM: floral buds at meiosis
FDR: false discovery rate
GO: Gene Ontology
KOG: Eukaryotic Orthologous Groups
PMCs: pollen mother cells
PI: propidium iodide
RT: room temperature
SC: synaptonemal complex
SRA: Sequences Read Archives

## References

[1] Liu S, Yang Y, Wei F, Duan J, Braynen J, Tian B, Cao G, Shi G, Yuan J (2017). Autopolyploid leads to rapid genomic changes in *Arabidopsis thaliana*. Theory Biosci.

[2] Chen Z (2010). Molecular mechanisms of polyploidy and hybrid vigor. Trends Plant Sci.

[3] Cifuentes M, Grandont L, Moore G, Chèvre AM, Jenczewski E (2010). Genetic regulation of meiosis in polyploid species: new insights into an old question. New Phytol.

[4] Abel S, Becker HC (2007). The effect of autopolyploidy on biomass production in homozygous lines of *Brassica rapa* and *Brassica oleracea*. Plant Breed.

[5] Li X, Yu E, Fan C, Zhang C, Fu T, Zhou Y (2012). Developmental, cytological and transcriptional analysis of autotetraploid *Arabidopsis*. Planta.

[6] Wang S, Chen W, Yang C, Yao J, Xiao W, Xin Y, Qiu J, Hu W, Yao H, Ying W, Fu Y, Tong J, Chen Z, Ruan S, Ma H (2016). Comparative proteomic analysis reveals alterations in development and photosynthesis-related proteins in diploid and triploid rice. BMC Plant Biol.

[7] Mayrose I, Zhan S, Rothfeis CJ, Magnuson-Ford K, Barker MS, Rieseberg LH, Otto SP (2011). Recently formed polyploid plants diversify at lower rates. Science.

[8] Soltis DE, Segovia-Salcedo MC, Jordon-Thaden I, Majure L, Miles NM, Mavrodiev EV, Mei W, Cortez MB, Solies PS, Gitzendanner MA (2014). Are polyploids really evolutionary dead-ends (again)? A critical reappraisal of Mayrose et al. (2011). New Phytol.

[9] Mayrose I, Zhang S, Rothfels CJ, Arrigo N, Barker MS, Rieseberg LH, Otto SP (2015). Methods for studying polyploid diversification and the dead end hypothesis: a reply to Soltis et al. (2014). New Phytol.

[10] Libeau P, Durandet M, Granier F, Marquis C, Berthome R, Renou JP, Taconnat-Soubirou L, Horlow C (2011). Gene expression profiling of *Arabidopsis* meiocytes. Plant Biol.

[11] Ma H (2005). Molecular genetic analyses of microsporogenesis and microgametogenesis in flowering plants. Annu Rev Plant Biol.

[12] Grandont L, Jenczewski E, Lloyd A (2013). Meiosis and its deviations in polyploid plants. Cytogenet Genome Res.

[13] Kumar G, Dwivedi K (2014). Induced polyploidization in *Brassica Campestris* L (*Brassicaceae*). Tsitol Genet.

[14] Wu J, Shahid MQ, Guo H, Yin W, Chen Z, Wang L, Liu X, Lu Y (2014). Comparative cytological and transcriptomic analysis of pollen development in autotetraploid and diploid rice. Plant Reprod.

[15] Chen L, Shahid MQ, Wu J, Chen Z, Wang L, Liu X (2018). Cytological and transcriptome analyses reveal abrupt gene expression for meiosis and saccharide metabolisms that associated with pollen abortion in autotetraploid rice. Mol Genet Genom.

[16] Chen C, Farmer AD, Langley RJ, Mudge J, Crow JA, May GD, Juntley J, Smith AG, Retzel EF (2010). Meiosis-specific gene discovery in plants: RNA-Seq applied to isolated *Arabidopsis* male meiocytes. BMC Plant Biol.

[17] Yang H, Lu P, Wang Y, Ma H (2011). The transcriptome landscape of *Arabidopsis* male meiocytes from high-throughput sequencing: the complexity and evolution of the meiotic process. Plant J.

[18] Braynen J, Yang Y, Wei F, Cao G, Shi G, Tian B, Zhang X, Jia H, Wei X, Wei Z (2017). Transcriptome analysis of floral buds deciphered an irregular course of meiosis in polyploid *Brasscia rapa*. Front Plant Sci.

[19] Albertin W, Brabant P, Catrice O, Eber F, Jenczewski E, Chevre AM, Thiellement H (2005). Autopolyploidy in cabbage (*Brasscia oleracea* L.) does not alter significantly the proteomes of green tissues. Proteomics.

[20] Ng DW, Zhang C, Miller M, Shen Z, Briggs SP, Chen ZJ (2012). Proteomic divergence in *Arabidopsis* autopolyploids and allopolyploids and their progenitors. Heredity.

[21] An F, Fan J, Li J, Li Q, Li K, Zhu W, Wen F, Carvalho LJ, Chen S (2014). Comparison of leaf proteomes of cassava (*Manihot esculenta Crantz*) cultivar NZ199 diploid and autotetraploid genotypes. PLoS ONE.

[22] Yan L, Fan G, Deng M, Zhao Z, Dong Y, Li Y (2017). Comparative proteomic analysis of autotetraploid and diploid *Paulownia tomentosa* reveals proteins associated with superior photosynthetic characteristics and stress adaptability in atuotetraploid *Paulownia*. Physiol Mol Biol Plants.

[23] Nicolas SD, Le Mignon G, Eber F, Coriton O, Monod H, Clouet V, Huteau V, Lostanlen A, Delourme R, Chalhoub B, Ryder CD, Chevre AM, Jenczewski E (2007). Homeologous recombination plays a major role in chromosome rearrangements that occur during meiosis of *Brassica napus* haploids. Genetics.

[24] Bomblies K, Higgins JD, Yant L (2015). Meiosis evolves: adaptation to external and internal environments. New Phytol.

[25] Lloyd A, Bomblies K (2016). Meiosis in autopolyploid and allopolyploid *Arabidopsis*. Curr Opin Plant Biol.

[26] Ramsey J, Schemske DW (2002). Neopolyploidy in flowering plants. Annu Rev Plant Biol.

[27] Comai L (2005). The advantages and disadvantages of being polyploid. Nat Rev Genet.

[28] Deng Z, Wang T (2007). OsDMC1 is required for homologous pairing in *Oryza sativa*. Plant Mol Biol.

[29] Nonomura K, Nakano M, Eiguchi M, Suzuki T, Kurata N (2006). PAIR2 is essential for homologous chromosome synapsis in rice meiosis I. J Cell Sci.

[30] He Y, Wang C, Higgins JD, Yu J, Zong J, Lu P, Zhang D, Liang W (2016). MEIOTIC F-BOX is essential for male meiotic DNA double-strand break repair in rice. Plant Cell.

[31] Xue Z, Li Y, Zhang L, Shi W, Zhang C, Feng M, Zhang F, Tang D, Yu H, Gu M, Cheng Z (2016). OsMTOPVIB promotes meiotic DNA double-strand break formation in rice. Mol Plant.

[32] Cordoba-Canero D, Roldan-Arjona T, Ariza RR (2011). *Arabidopsis* ARP endonuclease functions in a branched base excision DNA repair pathway completed by LIG1. Plant J.

[33] Huylenbroeck JM, Riek JD, Loose MD (2000). Genetic relationships among *Hibiscus syriacus*, *Hibiscus sinosyriacus* and *Hibiscus paramutabilis* revealed by AFLP, morphology, and ploidy analysis. Genet Resour Crop Evol.

[34] Mizukami Y, Fischer RL (2000). Plant organ size control: AINTEGUMENTA regulates growth and cell numbers during organogenesis. Proc Natl Acad Sci USA.

[35] Hu Y, Xie Q, Chua N (2003). The *Arabidopsis* auxin-inducible gene ARGOS controls lateral organ size. Plant Cell.

[36] Wang B, Sang Y, Song J, Gao X, Zhang X (2009). Expression of rice OsARGOS gene in *Arabidopsis* promotes cell division and expansion and increases organ size. J Genet Genom.

[37] Miller M, Zhang C, Chen ZJ (2012). Ploidy and hybridity effects on growth vigor and gene expression in *Arabidopsis thaliana* hybrids and their parents. G3.

[38] Marmagne A, Brabant P, Thiellement H, Alix K (2010). Analysis of gene expression in resynthesized *Brassica napus* allotetraploids: transcriptional changes do not explain differential protein regulation. New Phytol.

[39] Zhuang W, Gao Z, Wang L, Zhong W, Ni Z, Zhang Z (2013). Comparative proteomic and transcriptomic approaches to address the active role of GA_4_ in Japanese apricot flower bud dormancy release. J Exp Bot.

[40] Lan P, Li W, Schmidt W (2012). Complementary proteome and transcriptome profiling in phosphate-deficient *Arabidopsis* roots reveals multiple levels of gene regulation. Mol Cell Proteom.

[41] Nakaminami K, Matsui A, Nakagami H, Minami A, Nomura Y, Tanaka M, Morosawa T, Ishida J, Takahash S, Uemura M, Shirasu K, Seki M (2014). Analysis of differential expression patterns of mRNA and protein during cold-acclimation and de-acclimation in *Arabidopsis*. Mol Cell Proteom.

[42] Dong Y, Deng M, Zhao Z, Fan G (2016). Quantitative proteomic and transcriptomic study on autotetraploid paulownia and its diploid parent reveal key metabolic processes associated with paulownia autotetraploidization. Front Plant Sci.

[43] Vogel C, Marcotte EM (2012). Insights into the regulation of protein abundance from proteomic and transcriptomic analyses. Nat Rev Genet.

[44] Ye J, Zhang Z, Long H, Zhang Z, Hong Y, Zhang X, You C, Liang W, Ma H, Lu P (2015). Proteomic and phosphoproteomic analyses reveal extensive phosphorylation of regulatory proteins in developing rice anthers. Plant J.

[45] Bolanos-Villegas P, Xu W, Martínez-García M, Pradillo M, Wang Y. Insights into the role of ubiquitination in meiosis: fertility, adaptation and plant breeding. The *Arabidopsis* Book. 2018; 2018(16). 10.1199/tab.0187 PMC6501859 31068764

[46] Eloy NB, Gonzalez N, Van Leene J, Maleux K, Vanhaeren H, De Milde L, Dhondt S, Vercruysse L, Witters E, Mercier R, Cromer L, Beemster GT, Remaut H, Van Montagu MC, De Jaeger G, Ferreira PC, Inzé D (2012). SAMBA, a plant-specific anaphase-promoting complex/cyclosome regulator is involved in early development and A-type cyclin stabilization. Proc Natl Acad Sci USA.

[47] Murakami H, Keeney S (2014). Temporospatial coordination of meiotic DNA replication and recombination via DDK recruitment to replisomes. Cell.

[48] Lackner DH, Schmidt MW, Wu S, Wolf DA, Bähler J (2012). Regulation of transcriptome, translation, and proteome in response to environmental stress in fission yeast. Genome Biol.

[49] Shemesh-Mayer E, Ben-Michael T, Rotem N, Rabinowitch HD, Doron-Faigenboim A, Kosmala A, Perlikowski D, Sherman A, Kamenetsky R (2015). Garlic (*Allium sativum* L.) fertility: transcriptome and proteome analyses provide insight into flower and pollen development. Front Plant Sci.

