# Supplementary material for: Cytological and proteomic analyses of floral buds reveal an altered atlas of meiosis in autopolyploid Brassica rapa
Source: Cell Biosci. 2019 Jun 17;9:49. doi: 10.1186/s13578-019-0313-z (PMC6580506; doi:10.1186/s13578-019-0313-z)
Supplement: Supplementary file 2 — Additional file 2: Fig. S1. Statistics of abnormal meiotic course in autotetraploid B. rapa. Fig. S2. Immunolocalization of γH2AX in diploid and autotetraploid B. rapa. Fig. S3. Characterization of proteomics data. Fig. S4. Reproducibility analysis of three biological replicates by Pearson correlation coefficient. Fig. S5. Gene ontology classification and subcellular localization of identified proteins. Fig. S6. Expression patterns of 12 DEPs associated with phosphorylation in diploid and autotetraploid B. rapa. Fig. S7. KOG function classification of identified proteins. Fig. S8. Predicted protein–protein interaction network of meiosis-related DEPs in autotetraploid B. rapa. Fig. S9. Correlation analysis for the identified 305 DEPs and their transcriptome. [file 13578_2019_313_MOESM2_ESM.docx]

**Cytological and proteomic analyses of** **floral buds reveal an altered atlas of meiosis in polyploid *Brassica rapa***

Yan Yang ^1,2^, Fang Wei ^1,2^**^*^**, Janeen Braynen ^2,3^, Xiaochun Wei ^3^, Baoming Tian ^1^**^*^**, Gongyao Shi ^1^, Gangqiang Cao ^1^, Jiachen Yuan ^2^, Xiaowei Zhang^3^

*1. School of Agricultural Sciences, Zhengzhou University, Zhengzhou, Henan 450001,P.R.China*

*2. School of Life Sciences,* *Zhengzhou University, Zhengzhou, Henan 450001,P.R.China*

*3.* *Institute of Horticultural Research, Henan Academy of Agricultural Sciences, Zhengzhou, Henan 450002, P.R .China*

***Corresponding authors:** Fang Wei, E-mail:[fangwei@zzu.edu.cn](mailto:fangwei@zzu.edu.cn); and BaomingTian, E-mail: [tianbm@zzu.edu.cn](mailto:tianbm@zzu.edu.cn)


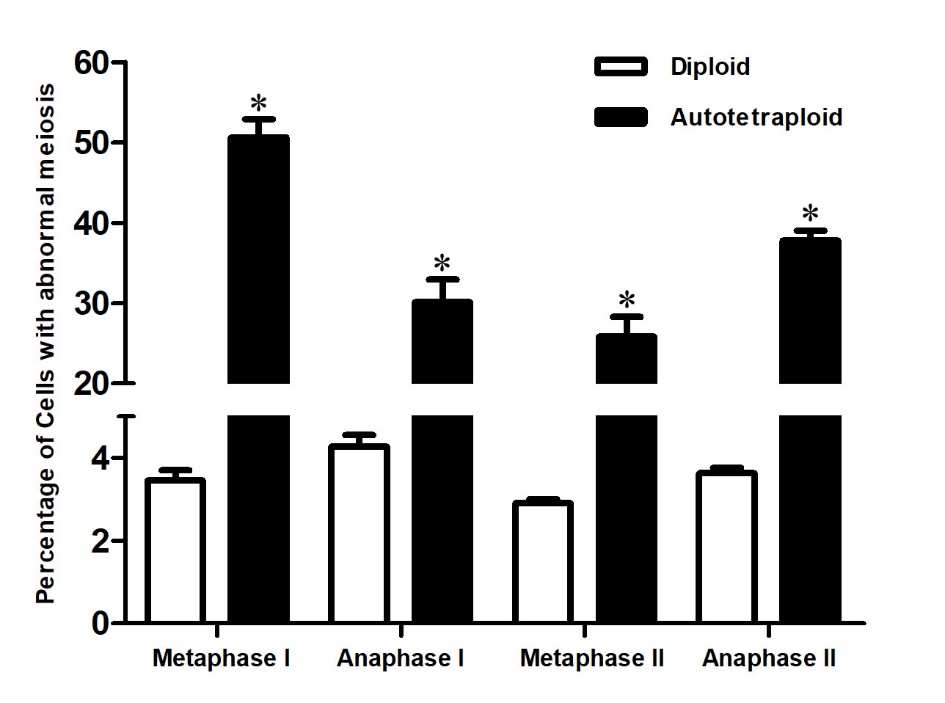


**Fig. S1** Statistics of abnormal meiotic course in autotetraploid *B. rapa*. Each bar represents the mean (±SE) of three independent biological replicates. The asterisk on the top indicates signiﬁcant differences (Student’s t test; P<0.05).


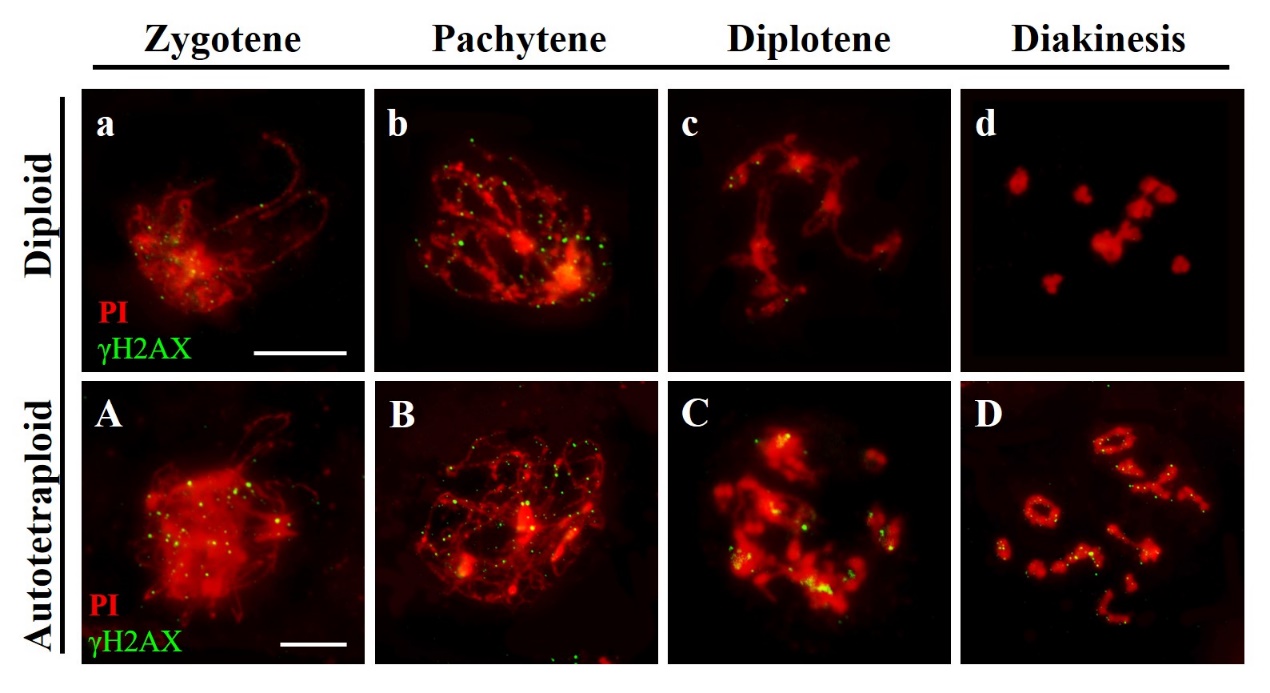


**Fig. S2** Immunolocalization of γH2AX in diploid and autotetraploid *B. rapa*. Chromosomal DNA was counterstained with PI (red), polyclonal γH2AX antibody was colored in green. (a, A) Zygotene, γH2AX foci were detected; (b, B) Pachytene, γH2AX foci were increased; (c, C) Diplotene, γH2AX foci disappeared in diploid *B. rapa* but occurred in autotetraploid *B. rapa*. (d, D) Diakinesis, γH2AX foci were still observed in autotetraploid *B. rapa*. Bar=5 μm.


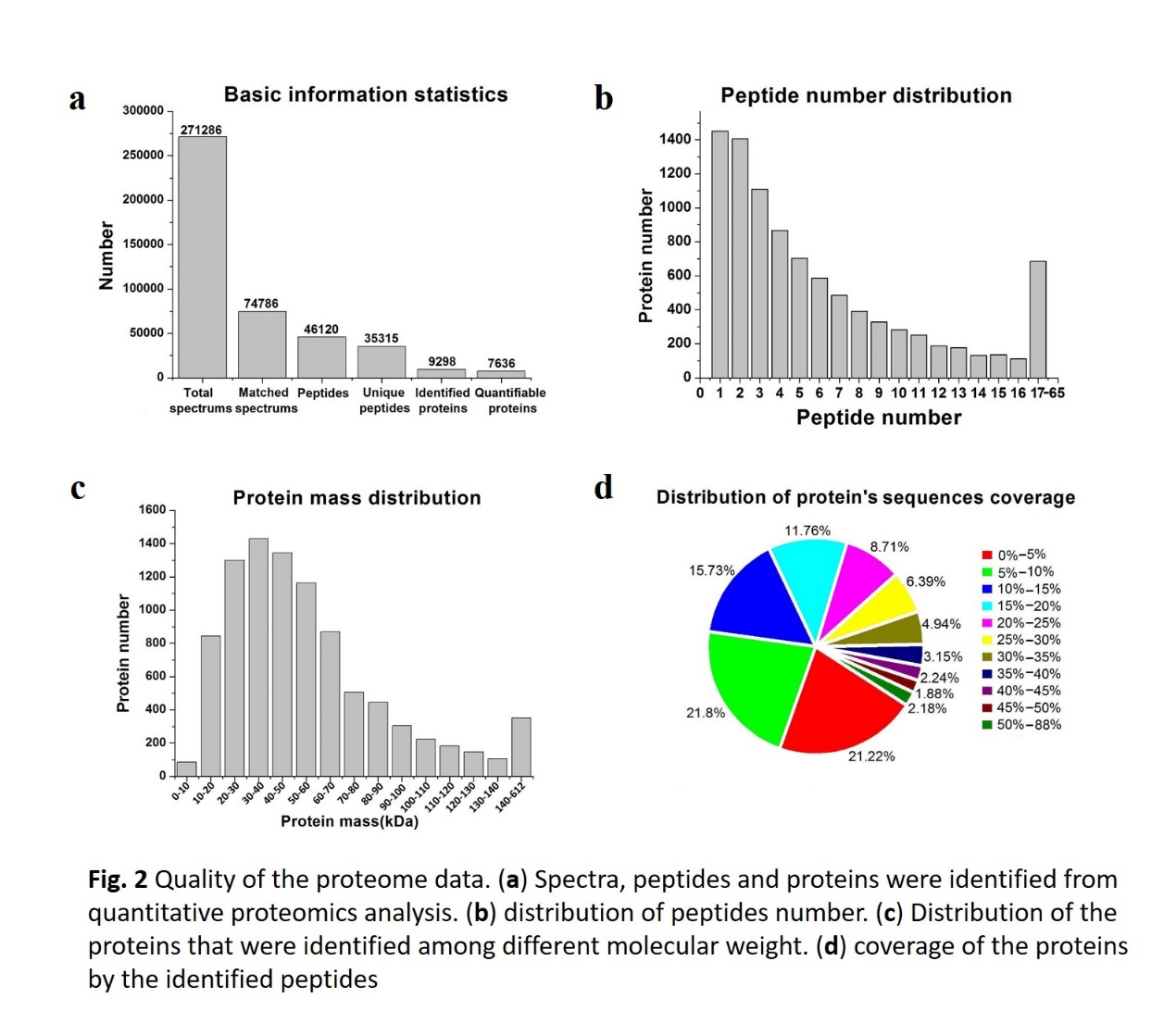


**Fig. S3** Characterization of proteomics data (a) Spectra, peptides and proteins were identified from quantitative proteomics analysis. (b) distribution of peptides number. (c) Distribution of the proteins that were identified among different molecular weight. (d) coverage of the proteins by the identified peptides


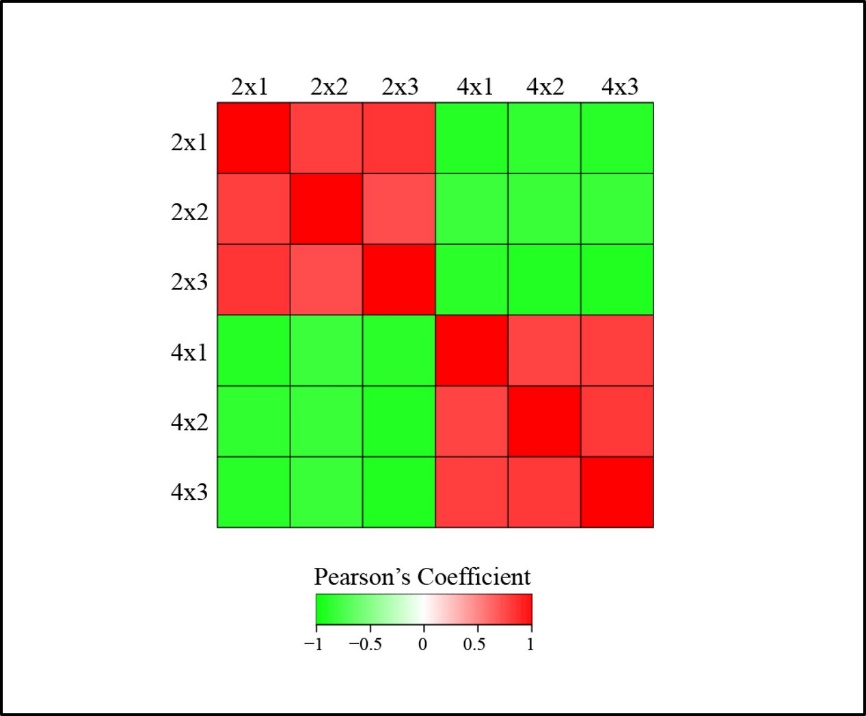


**Fig. S4** Reproducibility analysis of three biological replicates by Pearson correlation coefficient.


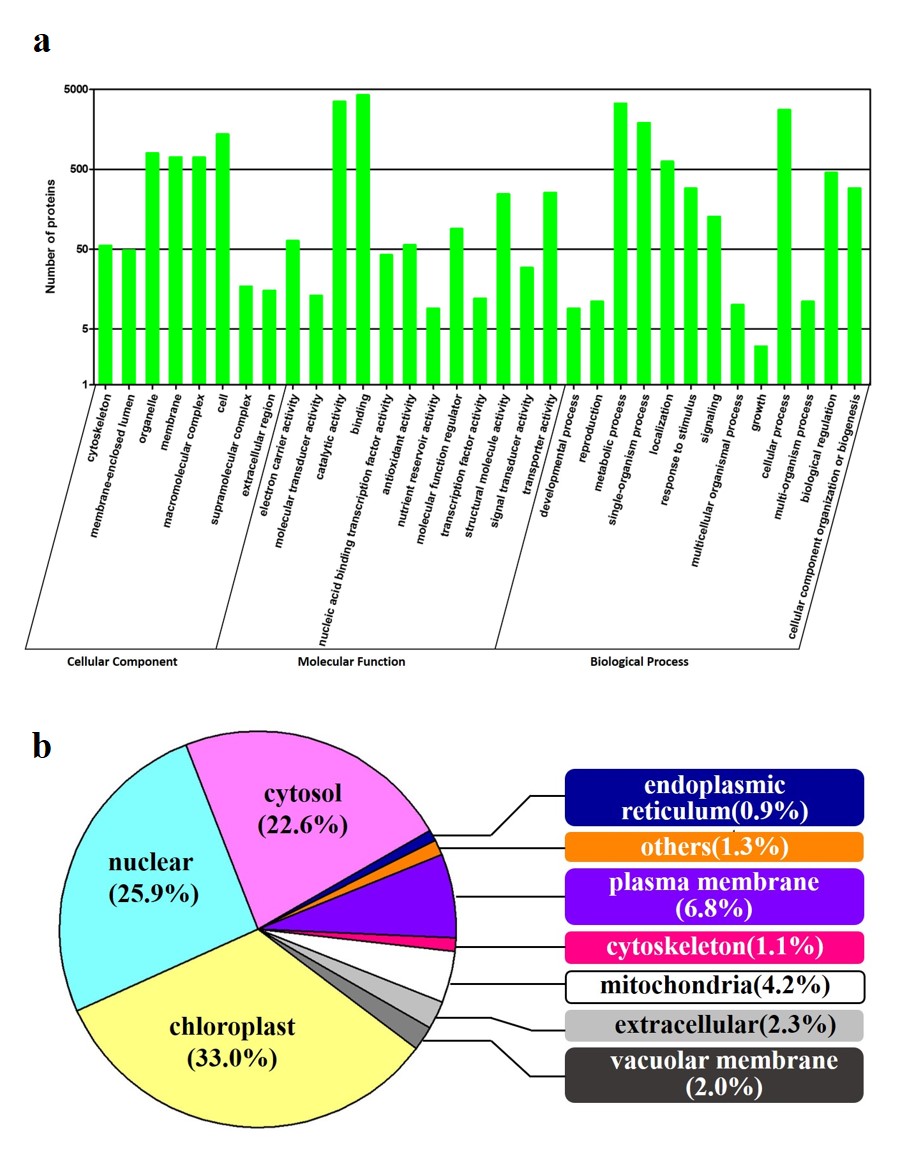


**Fig. S5** Gene ontology classification and subcellular localization of identified proteins.


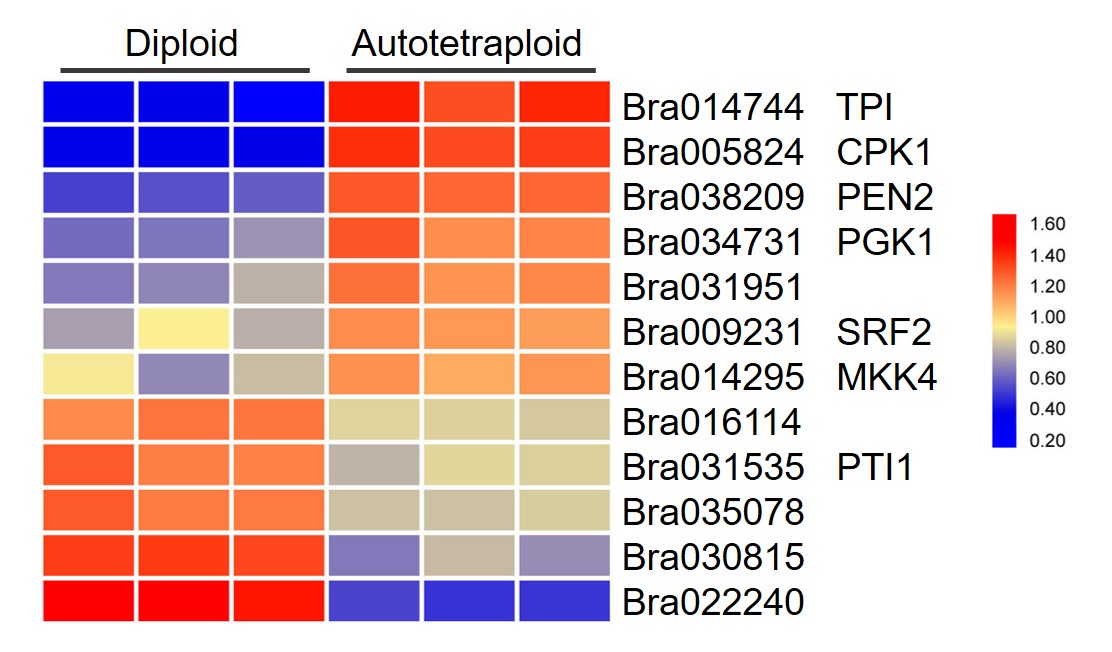


**Fig. S6** Expression patterns of 12 DEPs associated with phosphorylation in diploid and autotetraploid *B. rapa*.


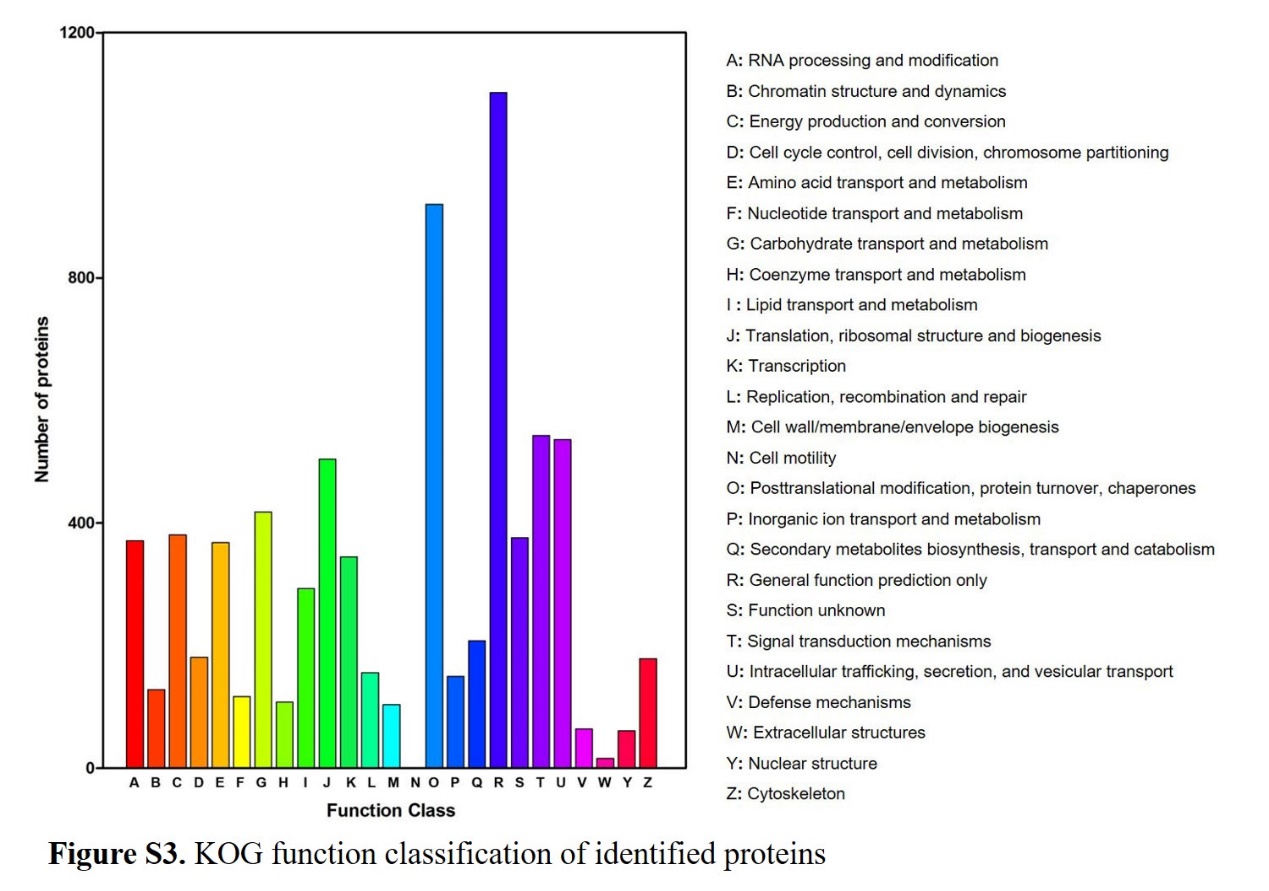


**Fig. S7** KOG function classification of identified proteins


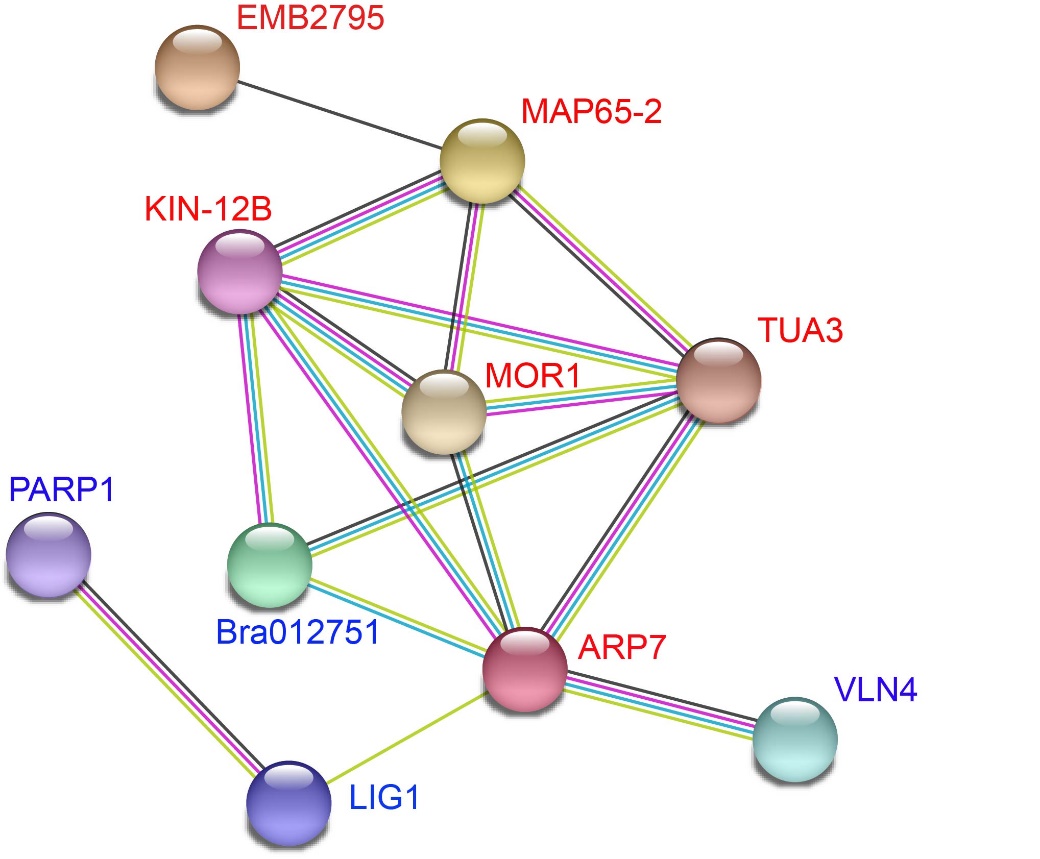


**Fig. S8** Predicted protein–protein interaction network of up-regulated (red) and down-regulated (blue) meiosis-related proteins in autotetraploid *B. rapa*.


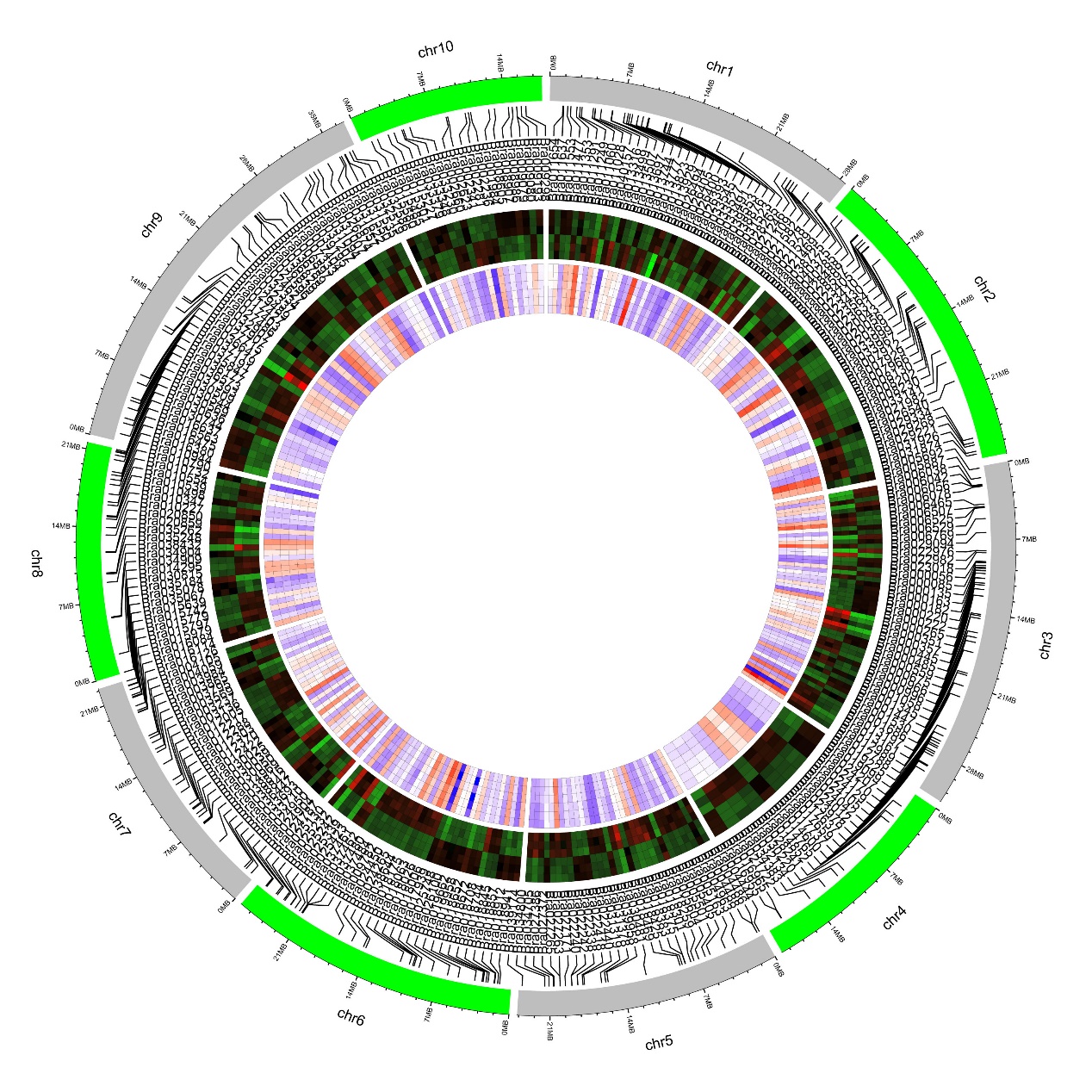


**Fig. S9** Correlation analysis for the identified 305 DEPs and their transcriptome
